# Multiple Rare Primary Malignancies: A Mixed Squamous Neuroendocrine Adenocarcinoma of the Cervix, Metastasized Carcinosarcoma and Extramammary Vulvar Paget’s Disease Case Report

**DOI:** 10.3390/medicina59050995

**Published:** 2023-05-21

**Authors:** Linas Andreika, Karolina Vankevičienė, Marija Plioplytė, Monika Bitinaitytė, Vilius Rudaitis

**Affiliations:** 1 Clinic of Obstetrics and Gynaecology, Institute of Clinical Medicine, Faculty of Medicine, Vilnius University, M. K. Čiurlionio Str. 21/27, LT-03101 Vilnius, Lithuania; vilius.rudaitis@santa.lt; 2 Faculty of Medicine, Vilnius University, M. K. Čiurlionio Str. 21/27, LT-03101 Vilnius, Lithuania; 3 Faculty of Medicine, Lithuanian University of Health Sciences, A.Mickevičiaus Str. 9, LT-44307 Kaunas, Lithuania; marija.plioplyte@stud.lsmu.lt (M.P.); monika.bitinaityte@stud.lsmu.lt (M.B.)

**Keywords:** carcinosarcoma, extramammary vulvar Paget’s disease, multiple primary malignancies, Müllerian carcinosarcoma, Malignant Mixed Müllerian Tumor, neuroendocrine adenocarcinoma

## Abstract

The occurrence of more than one primary malignant tumor in a single patient is rare. Multiple primary malignancies can pose difficulties in differential diagnosis between primary tumors and metastasis. Here, we present a case report with multiple primary malignancies. The patient is a 45-year-old female who was diagnosed with cervical mixed squamous neuroendocrine adenocarcinoma, metastasized carcinosarcoma and extramammary vulvar Paget’s disease. The patient was first diagnosed with a microinvasive squamous cervical carcinoma in situ. After a few months, the amputation of a small residual tumor and histological evaluation revealed an IA1-stage poorly differentiated (G3) mixed squamous and neuroendocrine cervical adenocarcinoma. After two years, the disease had progressed and biopsies from altered sites were taken. Histological diagnosis from an ulcerated vulvar region revealed extramammary vulvar Paget’s disease. A biopsy from vagina polyp revealed an earlier diagnosed mixed squamous and neuroendocrine cervical adenocarcinoma. However, histological diagnosis from an inguinal lymph node biopsy was unexpected and revealed carcinosarcoma. It indicated either the development of another primary malignancy, or an unusual spread of metastasis. Clinical presentation as well as diagnostic and treatment challenges are discussed in this case report. This case report shows that multiple primary malignancy cases are difficult to manage both for clinicians and the patient because the therapeutic options can become limited. This complex case was managed by a multidisciplinary team.

## 1. Introduction

The abundance of more than one primary malignant tumor occurring in a single patient is very low, statistically ranging between 0.73% and 11.7% [1]. There are risk factors that can lead to the development of multiple primary malignant tumors (MPMTs). These include genetics, exposure to carcinogens in food, environment, usage of tobacco and alcohol, hormonal changes, and imbalance of the immune system [2,3,4]. MPMTs can pose difficulties in differential diagnosis between a primary tumor and metastasis. This could be the case especially if a patient had already received treatment for another malignancy [5]. Additionally, with longer survival rates, cancer patients are exposed to higher risk of developing MPMTs due to the aftereffects of radiation and chemotherapy, continuous effects of genetics, or unhealthy habits [6,7]. This leads to a 2–17% chance of developing multiple primary malignancies [5]. Here, we present a patient with MPMTs. The case describes a woman diagnosed with a cervical neuroendocrine adenocarcinoma, metastasized carcinosarcoma and extramammary vulvar Paget’s disease.

## 2. Case Report

The patient was a 45-year-old woman with a past medical history of chronic pyelonephritis in her childhood and two previous pregnancies which resulted in one vaginal term delivery and one ectopic tubal pregnancy treated with laparoscopic tubectomy. Her family history was negative for oncological diseases.

In 2018, the patient started to complain about having post-coital bleeding that had lasted for 3 months. The Papanicolaou (PAP) smear revealed a high-grade intraepithelial lesion (HSIL). In October 2018, a large-loop excision of the transformation zone (LLETZ) of the cervix uteri was performed. Histology showed a microinvasive squamous cervical carcinoma with carcinoma in situ. The first conization was performed as a biopsy and showed microinvasive tumor masses in the coagulated edges of the specimen; therefore, it was impossible to determine the radicality of the procedure, and a secondary procedure, a trachelectomy, was indicated. Later, in January 2019, the amputation of a small residual tumor was performed, and histological evaluation revealed a IA1-stage poorly differentiated (G3) mixed squamous and neuroendocrine cervical carcinoma. The margins of the amputated mass were free of pathological changes in the histological specimen, and no lymphovascular invasion was detected, therefore, lymphadenectomy was not performed. No human papillomavirus (HPV) molecular signs were detected. The patient was sent to our tertiary level hospital for the upcoming surveillance after the primary diagnosis and treatment in the secondary level hospital. Follow up in 2019 showed no signs of residual disease. The PAP smear was normal with no signs of HPV infection. The patient suffered from mild vulvar itching, which was treated with corticosteroid ointments.

In September 2020, a relapse of the disease was suspected during routine gynecological examination due to observed pathological changes. While inspecting external genitalia, the vulvar region was ulcerated, inflamed, reddish, and tender. During examination with the speculum, the vaginal mucosa was atrophic, reddish, with a polypoid mass in the lower third of the vagina wall. The internal orifice was vaguely visible because of the amputated cervix. During the transvaginal ultrasound, no pathological changes were observed while examining the uterus or endometrium, except the right ovary with an 3.3 × 2.6 cm endometrioma. For more, the enlarged right inguinal lymph node was palpated. The patient had no complaints, and her general condition was satisfactory. There were no abnormalities in the complete blood count and comprehensive metabolic blood test panel, nor Ca-125, which was only slightly elevated above the upper limit of normal.

**Figure 1:**
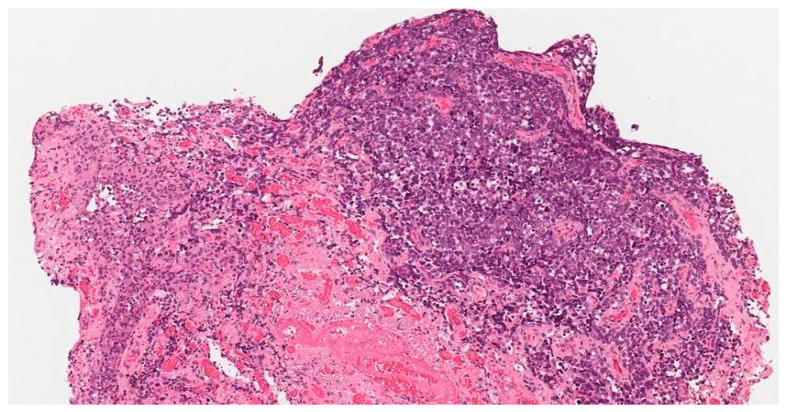
Biopsy from the vaginal polyp. Mixed, poorly differentiated G3 neuroendocrine adenocarcinoma.

**Figure 2:**
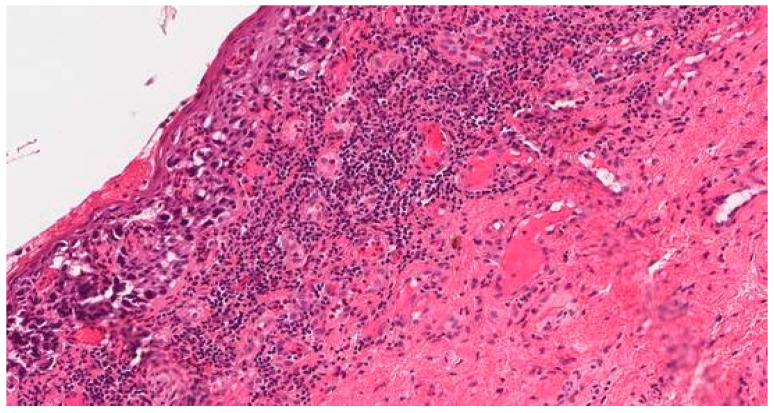
Biopsy from the injured vulvar tissues. Extramammary vulvar Paget’s disease.

**Figure 3:**
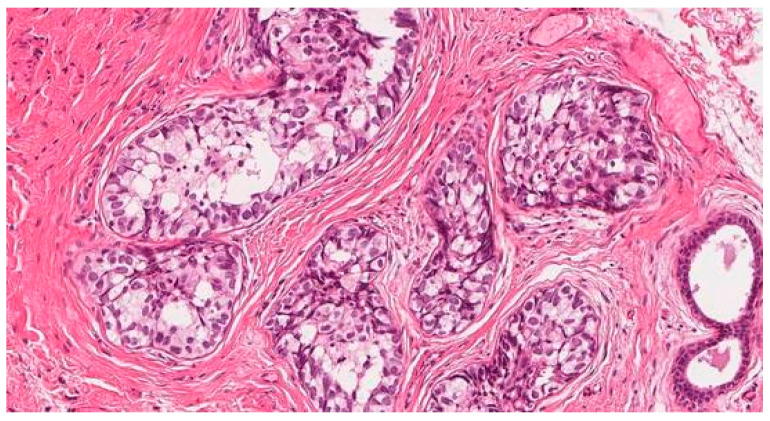
Biopsy from the injured vulvar tissues. Extramammary vulvar Paget’s disease. Ductal carcinoma-like lesions in situ.

To clarify the diagnosis, a PAP smear and biopsies from the vaginal wall polyp and ulcerated vulvar tissues were taken. The PAP smear showed signs of undifferentiated epithelial malignancy, with no signs of HPV. Biopsy from the vaginal wall polyp revealed histological changes with the corresponding immunoprofile of an earlier-diagnosed G3 mixed squamous and neuroendocrine cervical carcinoma (Figure 1). This biopsy confirmed the disease recurrence and spread in the genitalia. However, the histology from the vulvar biopsy revealed extramammary Paget’s disease with mucinous gland involvement and ductal carcinoma-like lesions in situ (Figure 2 and Figure 3).

**Figure 4:**
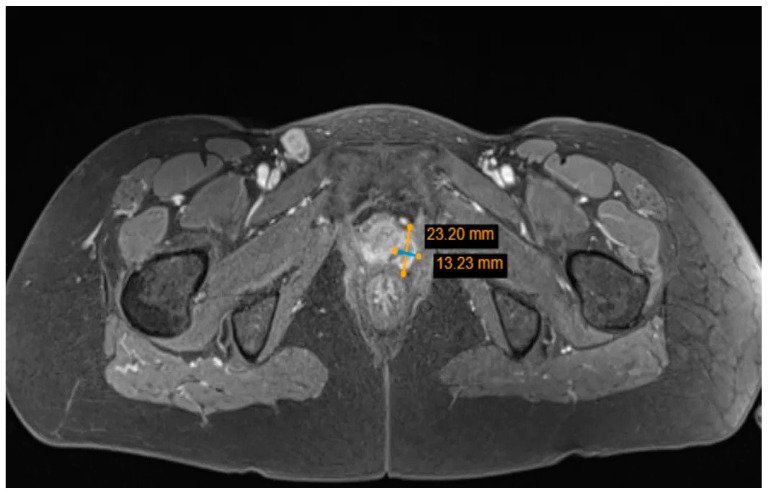
Pelvic MRI: tumor mass in the lower third of the vagina.

To evaluate the spread of the disease, pelvic and abdominal magnetic resonance imaging (MRI) was performed. MRI diagnosed a tumor mass without signs of extravaginal invasion in the lower third of the vagina (Figure 4), one right inguinal and one left rectovaginal lymph node pathologically affected, right ovarian endometrioma (this finding coincides with the ultrasound examination data), and a small amount of free fluid in the pelvis.

**Figure 5:**
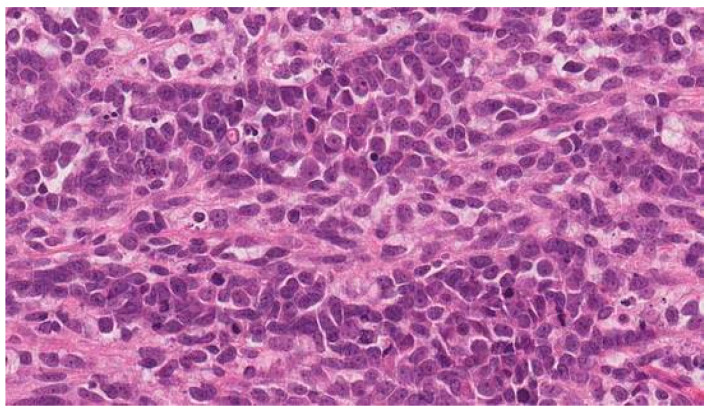
Biopsy from right inguinal lymph node: high-grade biphasic “primitive teratoid” malignant tumor–carcinosarcoma.

To determine the origin of the metastatic tumor, right inguinal lymph node biopsies under a spinal anesthesia were performed. However, the histology revealed a high-grade biphasic “primitive teratoid” malignant tumor–carcinosarcoma (Figure 5) and showed no signs of extramammary Paget’s disease or mixed squamous and neuroendocrine cervical adenocarcinoma (MANEC) metastases.

Without a clear histological diagnosis and a clear primary tumor site, a multidisciplinary team debated between surgical and systematic treatment. At that time, the tumor in the vagina was causing heavy bleeding and the only acceptable surgery would have been a pelvic exenteration without any guarantees of radical margins. After discussions with the patient, systemic chemotherapy treatment was proposed. The patient received seven chemotherapy cycles of Carboplatin with Paclitaxel and Bevacizumab every three weeks. Due to drug-induced thrombocytopenia, Carboplatin was replaced with Cisplatin, of which the patient received five cycles every three weeks. After the development of polyneuropathy, 17 cycles of Bevacizumab monotherapy were administered every three weeks. Overall, the patient received 29 cycles of first-line treatment.

In 2021, the MRI showed no signs of disease progression. A positive response was observed, and the systemic chemotherapeutic treatment was continued. The patient had no signs or symptoms of the disease.

**Scheme 1:**
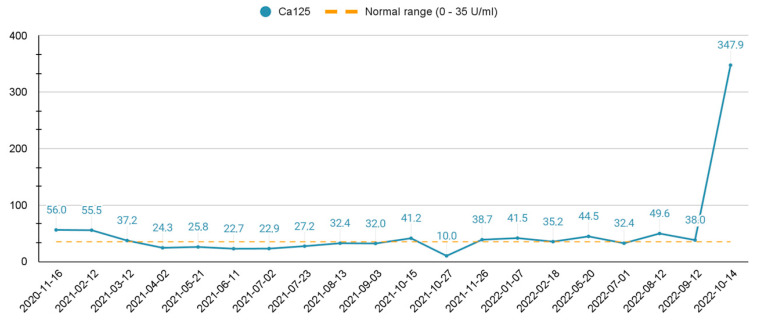
Ca-125 dynamic change over the course of the treatment.

In February 2022, a routine pelvis MRI control examination showed no signs of progression, although the abdominal MRI revealed a suspicious 11-mm lesion in the S4a segment of the liver, which was later specified as metastasis. In April 2022, the radiofrequency ablation of liver metastasis was performed. Bevacizumab monotherapy every three weeks was continued. In August 2022, pelvic MRI was performed and showed signs of relapse: local recurrence of a cervical tumor, nodes in the vagina with invasion of adjacent structures, and a pathological left external iliac lymph node. In suspicion of other remote metastasis, the patient was sent for an allergologist consultation and, afterwards, abdomen and chest CT scans were performed. The scans revealed multiple metastases in the liver (more than 50), Th6 and L3 vertebrae, and upper abdomen paraaortic and paracaval lymph nodes. Ca125 levels were increased; the dynamic changes are presented in Scheme 1. Due to the progression of disease, a multidisciplinary team of specialists recommended a second-line chemotherapeutic therapy treatment based on types of multiple primary malignancies, such as MANEC and carcinosarcoma. For the second-line treatment, it was decided to give three cycles every two weeks of Carboplatin with Gemcitabine and Denosumab. Only two full cycles were completed.

Despite the aggressive treatment regime, the disease progressed. Therefore, in September 2022, chemotherapy was discontinued, and symptomatic treatment was recommended. In October 2022, the patient started to complain of abdominal pain under the right upper quadrant lasting for one day and increased urination frequency. The urine color was dark, and analysis showed bilirubinuria. The ultrasound of the abdomen revealed ascites, multiple metastatic lesions in liver, and a dilated left hepatic duct, which had caused the mechanical jaundice. The multidisciplinary team concluded that surgical hepatic duct decompression was impossible. Specific chemotherapeutic or surgical treatments were not applicable due to liver failure and poor patient status of ECOG 3 (capable of only limited self-care; confined to bed or chair >50% of waking hours). Palliative care was recommended, including ascites drainage and appropriate medication treatment with antiemetics and analgesics. The patient was transferred to a palliative care hospice. The patient’s outcome was lethal.

## 3. Discussion

Multiple malignancies can be classified into synchronous and metachronous. Synchronous malignancies are diagnosed when a second primary tumor is found less than six months after the diagnosis of the first unrelated tumor. Metachronous malignancies are diagnosed in cases of more than six months separating the primary cancer detection and the second unrelated cancerous lesion [8]. In this case, the tumors are metachronous, because the second tumor diagnosis was established after a two-year period. Furthermore, it is difficult to differentiate whether a new lesion was a new primary malignant tumor, or the patient was presenting with an unusual spread of metastasis.

Synchronous malignancies are less common and account for approximately one-third of multiple primary gynecological malignancies, while metachronous cancers account for more than 60% [9]. According to research, metachronous malignancies, especially involving cervical cancer, are usually diagnosed at a younger age compared to synchronous cancers [10]. In this case, the patient was diagnosed with cervical cancer at the age of 41.

There are three etiology groups explaining the possible pathogenetic development mechanisms of multiple primary malignancies: genetic predisposition, previous cancer treatment, and environmental factors [11]. Patients with family history and mutations in mismatch repair genes are prone to developing gynecologic and colon cancer [12]. Patients who underwent cancer treatment or radiation therapy also have a higher possibility of a second primary malignancy [5]. Unhealthy diet, alcohol or tobacco use, unfavorable occupation conditions, viral infections, and other environmental factors can also contribute to the development of cancer [11]. Additionally, a previously diagnosed tumor could indicate that the patient is more susceptible to cancer development. To rule out any genetic predisposition, genetic testing should be performed. Nevertheless, in the case of a suspicion of a second primary malignancy, histological evaluation is necessary as well as comparison to the primary malignancy in order to rule out a diagnosis of metastasis [5].

In this case, the patient did not have a family history of oncological diseases and had never been treated with radiation therapy before. Thus, it could be deduced that environmental factors played a major role in the development of cancer for this patient.

The patient was first diagnosed with mixed squamous and neuroendocrine cervical adenocarcinoma (MANEC). MANEC is a mix of both neuroendocrine and exocrine cell populations [13]. It contributes to less than 1% of cervical cancers in women [14]. These cell populations can be characterized as individual tumors known as neuroendocrine carcinoma and adenocarcinoma. Thus, MANEC is diagnosed when each population accounts for at least 30% of the whole malignancy mass [15]. Therefore, diagnosing MANEC can be difficult, as it is hard to determine the percentage of abundant cell populations [13]. The pathogenesis of the MANEC is not clear, however, HPV is one of the most important cofactors in the development of cervical tumors. The role of HPV in cervical adenocarcinoma carcinogenesis found that the presence of HPV correlates to the patient’s age–infection and is more likely to be found in younger women [7]. In this clinical case, however, HPV viruses were not detected. In MANEC, the usual complaint is abnormal vaginal bleeding. At advanced stages, with the help of ultrasound, a mass in the lumen of the cervix could be detected [3]. A PAP smear is beneficial in detecting squamous cell carcinoma; however, the odds of detecting early precursors of adenocarcinoma are low [5]. Clinically, MANEC is considered an aggressive malignancy due to fast intravascular dissemination [13,16]. Its metastases usually spread to the ovaries, omentum, and peritoneum [17]. Cervical cancer metastases spread to the liver is rare, and the median interval between the primary diagnosis and the spread of metastases to the liver is approximately less than 40 months [18].

In this clinical case, diagnosis of MANEC was difficult. The patient complained of having post-coital bleeding, and a PAP smear revealed HSIL changes. However, after conization of the cervix uteri, the primary histological diagnosis was microinvasive squamous cervical carcinoma with carcinoma in situ. Only after the resection of the residual tumor was MANEC also detected, and the final histological diagnosis was poorly differentiated (G3) mixed squamous and neuroendocrine cervical carcinoma whose margins were free of pathological changes in the specimen. While histology is the golden standard of disease diagnosis and verification, in this case it took a long time because the foci of both tumors were unusually small, and thorough immunohistochemical staining was used after the diagnosis of disease progression. The diagnosis of neuroendocrine adenocarcinoma is based on finding at least two immunohistochemical markers [13]. Any subtype of adenocarcinoma is diagnosed when three characteristics are detected: increased mitotic activity, atypical nuclei, and apoptosis [14].

Imaging techniques such as MRI, CT, PET/MRI, and PET/CT are efficient in staging and detecting metastatic lymph nodes. PET/MRI is the most accurate one [15]. In this case, CT was performed first; however, it was not informative enough, as contrast was not used due to the patient’s sensitivity to iodine. As one of the most accurate diagnostic tests, MRI revealed pathological lymph node changes, which led to further examination of the patient.

After diagnosing extramammary vulvar Paget’s disease (EMPD), two possible metastatic etiologies of the lymph node were expected: metastasis from either vulvar EMPD, or cervical MANEC.

EMPD is a rare intraepithelial malignant condition originating mostly from apocrine cells. Intraepithelial adenocarcinoma usually affects the vulva, perianal area, and axilla [5]. The most commonly affected area is the vulva, accounting for 65% of EMPD cases. EMPD can be a secondary disease indicating other malignancies. Up to 15% of patients with EMPD are diagnosed with a carcinoma of the cervix, urethra, bladder, or rectum, and up to 25% of patients have a primary cutaneous adnexal carcinoma [6]. As in this clinical case, EMPD was observed together with MANEC.

Interestingly, biopsy of the lymph nodes revealed high-grade biphasic “primitive teratoid” malignant tumor–carcinosarcoma, which was neither EMPD nor MANEC. It indicated either the development of another primary malignancy, or an unusual spread of metastasis. Thus, the initial hypothesis for this patient could have been a Müllerian carcinosarcoma (MC), also known as a Malignant Mixed Müllerian Tumor (MMMT), which would have been the only primary tumor. Taking into consideration its tendency to metastasize widely, the MMMT could have manifested as numerous and morphologically different metastases.

An MMMT is a rare gynecological malignancy, accounting for less than 5% of all uterine corporeal malignant tumors. A MMMT originating from the uterine cervix is extremely rare, accounting for 0.005% of all cervical malignancies. Thus, the extension from the uterine corpus to the cervix must be excluded because MMMTs of the uterine corpus are more common [19,20]. An MMMT of the cervix or uterus can be distinguished based on the dominating tumor mass during clinical, instrumental, and histological specimen examination. It is a biphasic neoplasm consisting of both carcinomatous and sarcoma-like components [21]. When the primary location is unclear, a distinction can sometimes be made based on the epithelial component. However, most cervical and uterine MMMTs are microscopically indistinguishable [21,22]. The epithelial components include squamous cell carcinoma, adenoid basal carcinoma, adenoid cystic carcinoma, adenocarcinoma, serous adenocarcinoma, and poorly differentiated carcinoma [23]. The sarcomatous component might be homologous and heterologous. With MMMTs, the rarest sarcomatous component type includes neural differentiation. In our case, carcinosarcoma was diagnosed only from the lymph node biopsy. However, a cervical MMMT might be misdiagnosed as a primary tumor because the mesenchymal component was not represented in the biopsy specimen at that time.

The most common complaints and findings in patients diagnosed with carcinosarcoma include abnormal vaginal bleeding, palpable cervical mass, and abnormal PAP smear results [20]. In our case, when the relapse of the disease was suspected, the patient had heavy vaginal bleeding and a PAP smear which showed signs of undifferentiated epithelial malignancy.

Carcinosarcoma is very likely to metastasize with the usual metastatic spread pattern to pelvic organs and peritoneum [24]. Additionally, a MMMT metastasizes to abdominal organs such as liver, pancreas, lungs, and bones [25,26]. Liver metastases are extremely dangerous, resulting in a median survival of less than 8 months [27,28]. Metastases of a MMMT can vary due to different combinations of mesenchymal and epithelial parts, and may sometimes resemble other malignancies [29].

Because of the spread of the disease with distant metastases, systemic chemotherapeutic treatment was proposed to the patient. The first-line treatment for advanced or recurring cervical cancer is Carboplatin and Paclitaxel with an addition of Bevacizumab, which improves the outcome of the chemotherapy [30]. It is also indicated to administer this combination of medications every week, rather than every three weeks [31]. However, according to a meta-analysis of 10,000 patients (from 16 clinical trials), the use of Bevacizumab together with chemotherapy might increase the risk of a lethal outcome [32].

As the disease progressed notwithstanding the first-line chemotherapeutic treatment, a second-line treatment was proposed. Bevacizumab was replaced with Denosumab taking into account new metastases found in the vertebrae. Denosumab is a monoclonal antibody which is used for bone metastases treatment along with chemotherapy. This medication increases the interval of time between the spread of metastases to the bones and the onset of symptoms such as pressing to the spinal cord and pathological bone fractures [33].

The five-year survival rate of cervical cancer depends on the stage, metastases, patient’s condition, and patient’s age. The five-year relative survival rate for localized cervical cancer is 91.2%, for regional cervical cancer 59.8%, and for distant cervical cancer 18.9% [34]. The prognosis of cervical carcinosarcoma is uncertain. In a recent study, two year disease-free and overall survival rates in patients with stage I were higher than in those with stage II or greater (73% vs. 22% and 82% vs. 33%) [35]. In this clinical case, notwithstanding the recommended systemic chemotherapeutic treatment, the disease progressed, and the outcome was fatal. After detecting the first primary malignancy, the patient survived for four years.

## 4. Conclusions

Multiple primary malignancies are a rare pathology causing difficulties in diagnosis and treatment. Histological evaluation remains the key diagnostic tool, however, it can be difficult to differentiate between new primary malignancy and metastases, especially when a tumor such as an MMMT tends to form morphologically different metastases. Choosing the most applicable treatment option without an exact diagnosis is challenging. Without clarity regarding which tumor was primary, there was no certainty that the most suitable treatment regimen was chosen. Secondly, after the initiation of chemotherapy, the chance of detecting another malignancy which was not covered by the initial treatment option increases. Due to the treatment and diagnostic challenges of multiple primary malignancies, the patients’ survival rates are low, and outcomes are poor. In order to achieve the best outcomes for the patient diagnosed with multiple malignant tumors, the patient’s treatment should involve a multidisciplinary team approach.

## Data Availability

Data sharing is not applicable to this article.
